# Influence of the Nitrate-N to Ammonium-N Ratio on Relative Growth Rate and Crude Protein Content in the Duckweeds *Lemna minor* and *Wolffiella hyalina*

**DOI:** 10.3390/plants10081741

**Published:** 2021-08-23

**Authors:** Finn Petersen, Johannes Demann, Dina Restemeyer, Andreas Ulbrich, Hans-Werner Olfs, Heiner Westendarp, Klaus-Jürgen Appenroth

**Affiliations:** 1 Faculty of Agricultural Sciences and Landscape Architecture, University of Applied Sciences Osnabrück, Am Krümpel 31, 49090 Osnabrück, Germany; johannes.demann@hs-osnabrueck.de (J.D.); dina.restemeyer@hs-osnabrueck.de (D.R.); a.ulbrich@hs-osnabrueck.de (A.U.); h-w.olfs@hs-osnabrueck.de (H.-W.O.); h.westendarp@hs-osnabrueck.de (H.W.); 2 Matthias-Schleiden-Institute-Plant Physiology, University of Jena, Dornburger Str. 159, 07743 Jena, Germany; klaus.appenroth@uni-jena.de

**Keywords:** amino acids, biomass production, cultivation, Lemnaceae, nutrient medium, uptake, water lentils, yield

## Abstract

In order to produce protein-rich duckweed for human and animal consumption, a stable cultivation process, including an optimal nutrient supply for each species, must be implemented. Modified nutrient media, based on the N-medium for duckweed cultivation, were tested on the relative growth rate (RGR) and crude protein content (CPC) of *Lemna minor* and *Wolffiella hyalina*, as well as the decrease of nitrate-N and ammonium-N in the media. Five different nitrate-N to ammonium-N molar ratios were diluted to 10% and 50% of the original N-medium concentration. The media mainly consisted of agricultural fertilizers. A ratio of 75% nitrate-N and 25% ammonium-N, with a dilution of 50%, yielded the best results for both species. Based on the dry weight (DW), *L. minor* achieved a RGR of 0.23 ± 0.009 d^−1^ and a CPC of 37.8 ± 0.42%, while *W. hyalina*’s maximum RGR was 0.22 ± 0.017 d^−1^, with a CPC of 43.9 ± 0.34%. The relative protein yield per week and m^2^ was highest at this ratio and dilution, as well as the ammonium-N decrease in the corresponding medium. These results could be implemented in duckweed research and applications if a high protein content or protein yield is the aim.

## 1. Introduction

A growing world population, with an increasing demand for protein, will necessitate the efficient and increased production of food and animal feed. By 2050, the predicted global population is expected to increase to 9.5 billion, resulting in a rising global demand for protein of up to 78% under different scenarios [1]. In order to handle this challenge, cultivating plants with a high protein content is a promising option. One of the possible candidates is duckweed. However, for this purpose, duckweeds will need to be cultivated under standardized, large-scale conditions. 

Duckweeds are an aquatic plant family (*Lemnaceae*), which have been gaining increased attention as an option for human nutrition and animal feeding. Several studies showed the potential of certain duckweed species as a nutrient source [2,3,4]. This is due to high relative growth rates (RGR) [5,6], a protein content comparable to soybeans, and, in accordance with WHO recommendations, an amino acid composition suitable for humans. The species *Wolffiella hyalina* and *Wolffia microscopica*, in particular, have been recommended for human nutrition [2,3]. Additionally, the nutritional values and proportions within the duckweeds can be modified by changing the cultivation conditions [2,7,8].

Several duckweed species have been tested as feed for animals, such as chickens, piglets and fish [9,10]. High contents of the essential amino acids lysine and methionine make some duckweed species, such as *W. hyalina*, an interesting substitute for today’s foremost feed protein source soybean. Gwaze and Mwale [11] compiled several studies, which tested duckweed in pig nutrition. The replacement of soybean meal by 40% duckweed in the feeding rations of young piglets (0 to 10 days old) led to the highest average daily gain in body weight compared to the control of 100% soybean meal [12]. Nguyen and Ogle [13] showed that replacing 75% of roasted soybeans with *Lemna minor* in 5 to 15 week old Tau Vang chickens resulted in weight gain and a feed conversion optimum. 

Such promising studies have led to the challenge of yielding high quantities of protein-rich duckweed biomass from a standardized, large-scale production process to incorporate duckweed in the food and feed industry. To economically operate such a system, inexpensive and easily available resources should be used. Moreover, an optimal nutrient supply for each duckweed species must be identified. 

According to Appenroth et al. [14,15] no other medium supports faster growth of duckweeds than the N-medium. However, its only nitrogen source is nitrate, but different studies have emphasised the preferential uptake of ammonium over nitrate [16,17,18,19]. Little is known about the effect of different nitrate to ammonium ratios on the growth rate and nutritional components of duckweeds.

**Figure 1:**
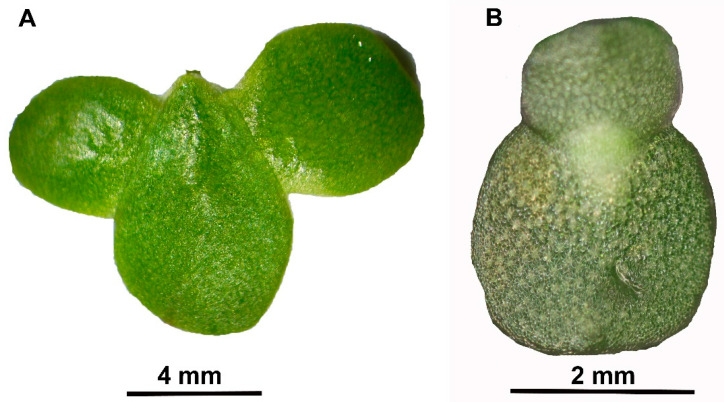
Investigated duckweed species. (**A**) *Lemna minor*, clone 9441. Mother frond (centre) is bearing two daughter fronds. (**B**) *Wolffiella hyalina*, clone 9525. Mother frond (bottom) is bearing a single daughter frond. Photos provided by Dr. K. Sowjanya Sree, Central University of Kerala, India.

The aim of our research was to examine how five different nitrate-N to ammonium-N ratios in modified N-media [14,15] affected the RGR, crude protein content (CPC), and relative protein yield (RPY) of *L. minor* and *W. hyalina* (Figure 1). In order to minimize the inhibiting effect of algae on duckweed growth rate and biomass production, different steps of dilution were investigated. Additionally, the decrease of NO_3_^−^-N and NH_4_^+^-N concentrations in the media, due to N-uptake by the duckweeds, was measured. 

## 2. Materials and Methods

### 2.1. Plant Material and Cultivation

Two duckweed species, *Lemna minor* L. (clone 9441; Germany) and *Wolffiella hyalina* Delile Monod (clone 9525; India), were used for the experiments due to their fast growth rates and high protein contents. The plant material was obtained from the Duckweed Stock Collection of the Department of Plant Physiology, University of Jena, Germany. 

Experiments were carried out in a climate chamber (length × width × height: 4 × 3 × 3 m) at the campus of the University of Applied Sciences Osnabrück, Germany. The trials were conducted in black PE-vessels with a diameter of 24 cm, each containing 4 L of nutrient medium. All modified media used were based on the N-medium [14,15]. The following abbreviations for five different NO_3_^−^-N to NH_4_^+^-N ratios are used throughout the manuscript: [100-0] = 100% NO_3_^−^-N - 0% NH_4_^+^-N; [75-25] = 75% NO_3_^−^-N - 25% NH_4_^+^-N; [50-50] = 50% NO_3_^−^-N - 50% NH_4_^+^-N; [25-75] = 25% NO_3_^−^-N - 75% NH_4_^+^-N; and [0-100] = 0% NO_3_^−^-N - 100% NH_4_^+^-N. In preliminary experiments, it was observed that the growth of duckweed was disturbed by contamination of ubiquitous algae in the cultures. In order to minimize nutrient competition and growth inhibition of the duckweed due to algae and microorganism growth, several measures were implemented. Two different dilutions (10% and 50% of the original concentration) were used for all five NO_3_^−^-N to NH_4_^+^-N ratios, indicated by “/10” and “/50” following the ratios. Dilutions of 1% and 5% (/1; /5) were used in initial experiments with [100-0], but omitted in later experiments because of poor results. The temperature was kept at 20.4 ± 1.3 °C. S4W LED elements (SANlight GmbH, Bludenz, Austria), with a photosynthetically active radiation of 350 µmol m^−2^ s^−1^, were used as the light source. The photoperiod was set to 8 h of light and 16 h of darkness. 

The pH showed a minor increase throughout the experimental period, rising from pH 6.6 to a maximum value of 7.0 in the 50% dilutions and from 7.2 to a maximum value of 8.0 in the 10% dilutions. 

Six stock solutions, used for all five differently modified N-media, were mainly prepared with commercially available agricultural fertilizers and deionized water. The following products were used for preparing the stock solutions: Krista MKP, Calcinit, Krista K Plus (Yara GmbH and Co. KG, Dülmen, Germany), OCI Granular 2 (OCI NV, Amsterdam, Netherlands), potassium chloride, sodium molybdate dihydrate technical grade (AppliChem GmbH, Darmstadt, Germany), ammonium chloride p.a., calcium chloride dihydrate (Merck KGaA, Darmstadt, Germany), Borax, Mangaan (Horticoop, Bleiswijk, Netherlands), Epso Combitop (K+S AG, Kassel, Germany) and Ferty 72 (Planta Düngemittel GmbH, Regenstauf, Germany). The precise formulation for each stock solution is presented in Table 1.

The stock solutions were diluted with local tap water (see Table S1) to obtain the initial N-medium concentrations of 100%, as shown in Table 2.

Table 3 depicts the measured concentrations for nitrate-N and ammonium-N, as well as the corresponding electrical conductivity (EC), after dilution to the final concentrations of 1%, 5%, 10%, and 50% of the undiluted medium.

Pre-cultivation occurred for three days within each of the differently diluted and modified nutrient media in order to avoid the lag-phase effect on the RGR data. Experiments lasted for seven days and were conducted under non-axenic growth conditions. The vessels were placed in a randomized block design within the climate chamber. In order to start with a similar surface coverage of 60%, the initial fresh weight biomass of 2 g for *L. minor* and 1.5 g for *W. hyalina* was placed in the above described vessels. At the end of the experiment, the duckweeds were harvested with a metal sieve, rinsed with fresh tap water to remove the attached nutrient solution, blotted with a paper towel to remove attached water, and weighed.

### 2.2. Analytical Methods

The dry weight (DW) was determined from fresh weight by oven drying at 65 °C for 72 h. At time 0, four samples per species of the same fresh weight as the starting material were used to determine the DW at the beginning of the experiments. 

The RGR per day was calculated according to Equation (1) [5], using the values of the DW at the start (t0) and after seven days of cultivation (t7):

RGR = (lnDW_t7_ − lnDW_t0_)/(t7 − t0) (1)

where RGR is the relative increase of the DW per unit time of 1 day (d^−1^). The relative weekly yield (RY; g biomass obtained after one week of cultivation starting with 1 g) was calculated from the RGR using Equations (2) and (3):

lnDW_t7_ = lnDW_t0_ + RGR · (t7 − t0) (2)

RY = exp(lnDW_t7_) (3)

The RY (see Figure S1) was further used to calculate the RPY (g protein week^−1^ m^−2^) by multiplying it with the CPC and extrapolating it to one square metre, according to Equation (4):

RPY = RY · CPC/(0.0452 m^2^ · 100) (4)

where 0.0452 m^2^ is the cultivation area of the vessels used in the experiment.

Dried samples were ground and homogenized using a laboratory mill and stored for further analysis. The nitrogen content of the dried samples was determined by the Dumas method [20] using a FP628 (Leco, Saint Joseph, MI, USA), and was multiplied with the factor 6.25 to determine the CPC [2,21].

Nutrient solution samples were taken at the start (day 0) and end (day 7) of the experiment from each vessel, which were filtered (MN 619 EH, Machery Nagel GmbH and Co. KG, Düren, Germany) to remove particles and instantly frozen at −18 °C. The nitrate-N and ammonium-N concentrations in these samples were measured according to German standard methods [22,23] with a Lambda 25 UV/VIS Spectrometer (Perkin Elmer, Waltham, MA, USA). 

### 2.3. Statistics

All the data is based on four replicates, which are given as mean ± standard deviations. The data were analysed statistically by one-way ANOVA and Tukey’s post hoc test at 5% significance level, using the software program SPSS 25 (IBM, Armonk, NY, USA). Datasets fulfilled the one-way ANOVA postulates (including normal distribution and homogeneity of variance).

## 3. Results

### 3.1. Growth

**Figure 2:**
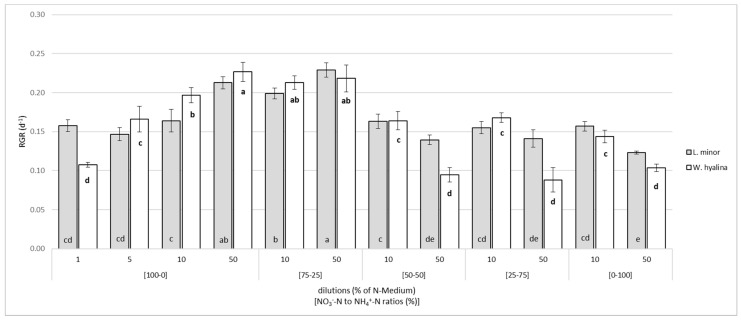
Relative growth rate (RGR; d^−1^), based on dry weight, for *Lemna minor* (grey shaded columns) and *Wolffiella hyalina* (white columns). Plants were cultivated for seven days in nutrient solutions with varying nitrate-N to ammonium-N ratios (from [100-0] to [0-100]) in different dilutions (1, 5, 10, and 50% of the undiluted N-medium). For the different nutrient media see Table 1, Table 2 and Table 3. Number of parallel samples n = 4. Different letters indicate significances within a species, based on one-way ANOVA test, Tukey *p* ≤ 0.05. Error bars indicate standard deviations.

The RGR was determined in dependence on the different nutrient media used (Figure 2). The highest value for *L. minor* was 0.23 ± 0.009 d^−1^ at [75-25]/50. The same RGR was determined for *W. hyalina* at [100-0]/50. The two most nitrate-rich ratios ([100-0] and [75-25]) showed an increasing RGR at higher nutrient concentrations (dilutions of 50%) compared to the 10% dilutions, which was significantly higher for *L. minor* in both ratios and for *W. hyalina* only in [100-0]. In these two ratios and dilutions, *W. hyalina* had a slightly higher RGR than *L. minor*, with the exception of [75-25]/50. This was contrary to when the ammonium concentration was increased. A significant drop of the RGR was observed for the ratios [50-50], [25-75], and [0-100] for *L. minor* compared to the 50% dilutions and for *W. hyalina* compared to the 10% and 50% dilutions, but it was more severe in the 50% dilutions. This decrease resulted in a minimum RGR of 0.09 ± 0.015 d^−1^ at [25-75]/50 for *W. hyalina*, while the decrease for *L. minor* (0.12 ± 0.002 d^−1^ at [0-100]/50) was less severe. *Lemna minor* achieved higher RGRs than *W. hyalina* at an overall lower level compared to the two most nitrate-rich ratios, except for [100-0]/1.

### 3.2. Crude Protein Content and Protein Yield

**Figure 3:**
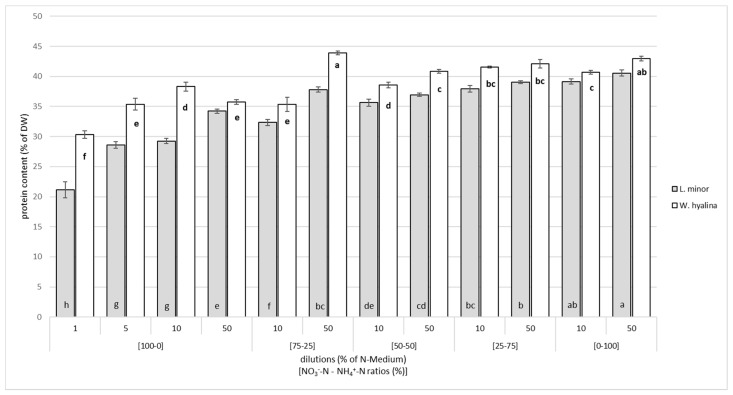
Crude protein content (% of DW) for *Lemna minor* (grey shaded columns) and *Wolffiella hyalina* (white columns), cultivated for seven days in nutrient solutions with different NO_3_^−^-N to NH_4_^+^-N ratios in different dilutions based on N-medium. For further explanations, see Figure 2.

The CPC increased in both duckweed species with increasing ammonium concentrations and a higher ammonium-N to nitrate-N ratio, but not significantly (Figure 3). The higher dilution of the nutrient media, i.e., lower nutrient concentrations, led to lower CPCs within each ratio. In general, *W. hyalina* had a higher CPC within each ratio and dilution compared to *L. minor*. The highest CPC in *L. minor* was reached at [0-100]/50 with 40.6 ± 0.48%, followed by 39.1 ± 0.43% at [0-100]/10. The maximum value measured for *W. hyalina* was 43.9 ± 0.34% at [75-25]/50, which is not significantly higher than the second highest CPC (43.0 ± 0.4%) at [0-100]/50. A minimum CPC of 21.1 ± 1.3% for *L. minor* and 30.3 ± 0.6% for *W. hyalina* were obtained in the ratio with the lowest concentration of nutrients available for the plants ([100-0]/1), which are significantly lower than the second lowest values for each species.

**Figure 4:**
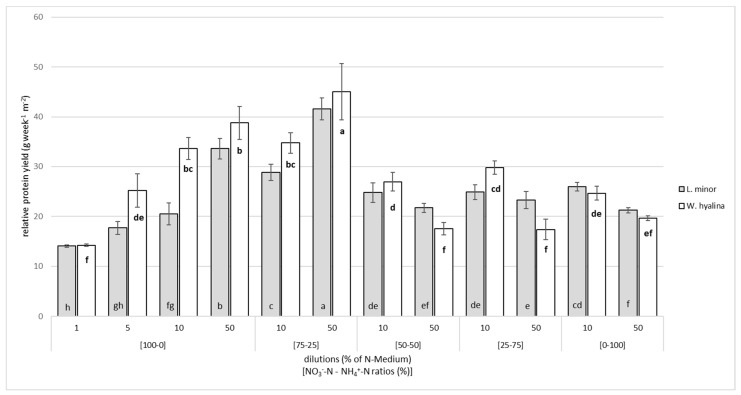
Relative protein yield, in grams per week and m^2^ (based on DW), for *Lemna minor* (grey shaded columns) and *Wolffiella hyalina* (white columns), cultivated for seven days in nutrient solutions with different NO_3_^−^-N to NH_4_^+^-N ratios in different dilutions based on N-medium. For further explanations, see Figure 2.

The highest RPY (g protein week^−1^ m^−2^) was obtained at [75-25]/50 for both species, with a significant difference from the second highest value (Figure 4). A total of 41.6 ± 2.2 g week^−1^ m^−2^ were harvested from *L. minor* and 45.0 ± 5.7 g week^−1^ m^−2^ from *W. hyalina.* A higher nutrient concentration in the ratios [100-0] and [75-25] led to a higher protein yield. *W. hyalina* yielded more protein than *L. minor* under these conditions. The protein yield significantly decreased with an ammonium concentration of 50% and more compared to [100-0]/50 and [75-25]/50 for *L. minor* and [75-25]/50 for *W. hyalina*. At dilutions of 50%, *W. hyalina* performed worse than *L. minor*, while the RPY for both species was slightly higher at 10% dilutions at an overall low level of less than 30 g week^−1^ m^−2^. The minimum RPYs of 14.1 ± 0.24 g week^−1^ m^−2^ for *L. minor* and 14.2 ± 0.28 g week^−1^ m^−2^ for *W. hyalina* were obtained in [100-0]/1. 

### 3.3. NO_3_^−^-N and NH_4_^+^-N Reduction in the Media

**Figure 5:**
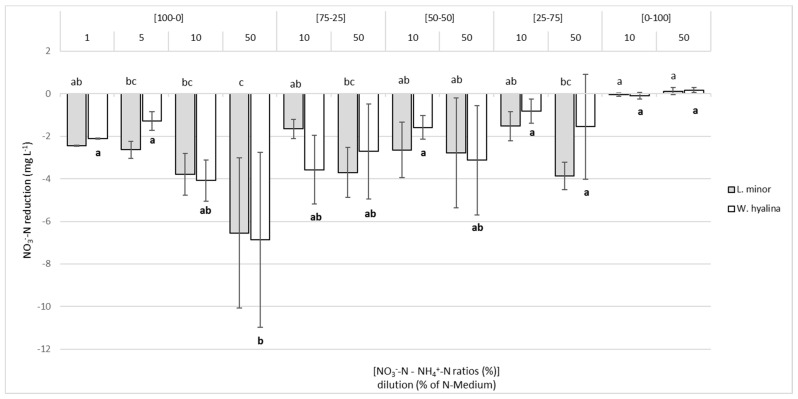
Decrease of the concentration of nitrate-N (mg L^−1^) in the nutrient media for *Lemna minor* (grey shaded columns) and *Wolffiella hyalina* (white columns), cultivated for seven days in nutrient solutions with varying NO_3_^−^-N to NH_4_^+^-N ratios in different dilutions. For further explanations, see Figure 2.

Figure 5 shows the total reduction of NO_3_^−^-N (mg L^−1^) for each ratio and dilution at day seven. In the nitrate-only medium, [100-0], the higher NO_3_^−^-N concentration led to a significantly higher absolute reduction in *L. minor*, but not in *W. hyalina*. The highest starting concentration of nitrate-N ([100-0]/50) led to the highest absolute decrease of nitrate-N, i.e., 6.5 mg L^−1^ and 6.9 mg L^−1^ for *L. minor* and *W. hyalina*, respectively. This corresponded to a relative reduction of 9.1% (*L. minor*) and 9.7% (*W. hyalina*). In general, higher nitrate-N concentrations resulted in a greater reduction, while an increasing NH_4_^+^-N concentration led to a decreasing nitrate-N reduction. These findings, however, were not significant. The maximal relative reduction of NO_3_^−^-N for *L. minor* was found at [25-75]/10 with 29.2% and for *W. hyalina* at [75-25]/10 with 29.6%. 

**Figure 6:**
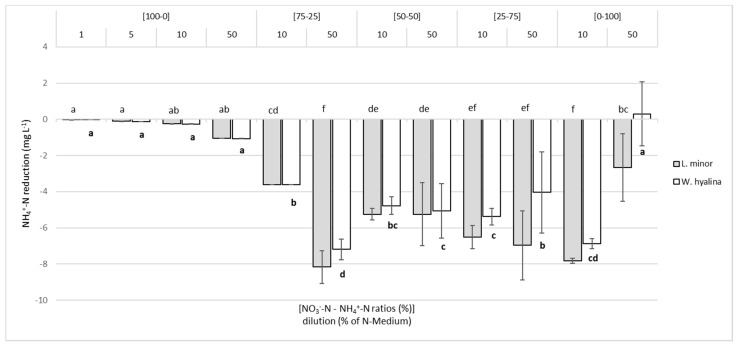
Decrease of the concentration of ammonium-N (mg L^−1^) in the nutrient media for *Lemna minor* (grey shaded columns) and *Wolffiella hyalina* (white columns), cultivated for seven days in nutrient solutions with varying NO_3_^−^-N to NH_4_^+^-N ratios in different dilutions. For further explanations, see Figure 2.

Figure 6 depicts the total reduction of NH_4_^+^-N (mg L^−1^) for each ratio and dilution at day seven. In the [100-0] nutrient media, NH_4_^+^-N was present only in minor concentrations, which were decreased almost completely by both duckweed species. This also applied to [75-25]/10. The highest total reduction values were 8.1 ± 0.9 mg L^−1^ for *L. minor* and 7.2 ± 0.5 mg L^−1^ for *W. hyalina* in the [75-25]/50 treatments. This corresponded to a relative reduction of 46.8% (*L. minor*) and 41.6% (*W. hyalina*). The total, as well as the relative reduction, was slightly higher for *L. minor* than for *W. hyalina*. A significant drop was evident in the ammonium-only solutions, with the highest total NH_4_^+^-N concentration ([0-100]/50), compared to the same dilution in the ratios [75-25], [50-50], and [25-75]. The relative reduction for *L. minor* was 4.1% of the initially available NH_4_^+^-N, while for *W. hyalina* no reduction at all occurred. However, decreasing the total NH_4_^+^-N concentration, but keeping the ratio of the two N sources constant, i.e., [0-100]/10, resulted in much higher uptake rates for *L. minor* (7.8 ± 0.14 mg L^−1^; 54% relative reduction) and *W. hyalina* (6.9 ± 0.27 mg L^−1^; 48% relative reduction).

## 4. Discussion 

The maximum RGR reached in this experiment was 0.23 d^−1^ for both species, which is lower compared to other studies. For *L. minor,* an RGR of 0.42 d^−1^ was reported, while *W. hyalina* had the highest RGR of all investigated species with a value of 0.519 d^−1^ [5]. The difference in the RGR was most likely caused by different growth conditions. Instead of an axenic in vitro set-up, both duckweed species in this study were cultivated under non-sterile conditions. The temperature was 5 °C lower and the photoperiod 16 h shorter, while the light intensity was 250 µmol m^−2^ s^−1^ higher compared to the experimental set-ups applied by Ziegler et al. [5]. These factors are possible explanations for the lower RGRs. 

Iatrou et al. [24] achieved a maximum growth rate of 0.14 d^−1^ for *L. minor* at an ammonium-N concentration of 31.9 mg L^−1^, using secondary treated wastewater. This was in agreement with our experimental results for the same species in the ratio of [50-50]/50 (RGR of 0.14 ± 0.009 d^−1^ at a NH_4_^+^-N concentration of 32.5 mg L^−1^). 

Caicedo et al. [18] observed that the highest RGR (0.3 d^−1^) in *Spirodela polyrhiza* was obtained at the lowest total ammonium concentrations (3.5–20 mg L^−1^ N; equal to ca. 0.25–1.4 mM) and assumed an optimum NH_4_^+^-N concentration was below 20 mg L^−1^. Zhang et al. [25] obtained the maximal RGR in *L. minor* at 3.5 mg L^−1^ ammonium-N. These data partly agree with our results concerning the total concentration of NH_4_^+^-N. High RGRs were obtained for the treatments [75-25]/10, with an initial NH_4_^+^-N concentration of 3.6 mg L^−1^, and [75-25]/50, with a concentration of 17.3 mg L^−1^. The 10% dilutions [50-50]/10, [25-75]/10, and [0-100]/10 had similar total NH_4_^+^-N starting values of 7.1, 10.7, and 14.4 mg L^−1^, respectively. However, the RGR was significantly lower in these three treatments for both species. It can be assumed that other factors, such as the ratio of nitrate to ammonium, had a certain impact on the RGRs of *L. minor* and *W. hyalina*. This is in agreement with the investigations of Mehrer and Mohr [26] and Hecht and Mohr [27] on *Sinapis alba* seedlings. The explained the detrimental effects of higher ammonium concentrations by identifying that ammonium accumulation is not well regulated by plants. The stimulation of ammonium assimilation by simultaneously applied nitrate appears to explain the nitrate-mediated ammonium tolerance. A similar mechanism exists in duckweeds, as shown recently in *Landoltia punctata* [28]. A minor fraction of ammonium as the nitrogen source seemed to stimulate duckweed growth, while proportions of 50% and higher had a growth inhibiting effect. 

Approximately six NO_3_^−^ transporters and four NH_4_^+^ transporters are involved in the uptake of N for *Arabidopsis thaliana*. Nitrate acts as a signalling molecule that triggers changes in the expression of genes, metabolism, and growth. Plants have evolved several NO_3_^−^ uptake systems to survive in the changing environment. While low affinity transporters are responsible for the uptake of a large amount of nitrate in the case of available high concentrations, high affinity transporters ensure plant survival in the presence of low nitrate concentrations [29]. Acquisition of ammonium from the aquatic environment is important, as this N source for plants may be the dominating form under certain conditions. While considerable progress was made in the last two decades, many aspects of the regulation of NH_4_^+^ uptake and metabolism are not yet well understood [30]. *Lemna minor* grown in an NH_4_NO_3_ (1:1 ratio between NH_4_^+^-N and NO_3_^−^-N) containing nutrient solution preferentially took up ammonium over nitrate. It was discovered that both roots and fronds take up nitrate and ammonium from the medium. At low N concentrations, the root-to-frond biomass ratio increased. This is advantageous for the plant at a morphological level, wherein there is a lower biomass investment per unit surface area for roots than for fronds [19]. Fang et al. [16] reported a preference in NH_4_^+^ uptake compared to NO_3_^−^ in *Landoltia punctata*. Turions of the duckweed *Spirodela polyrhiza* absorbed ^15^NH_4_^+^ much faster than ^15^NO_3_^−^ [31]. This was confirmed by our data, which showed that the average relative uptake rate of NH_4_^+^-N in almost all ratios and dilutions was higher than that of NO_3_^−^-N. 

A decrease of the ammonium concentration in nutrient media can be caused by plant uptake or by volatilization depending on the pH. With a pH value of 8 at 20 °C, less than 5% of the ammonium turns into NH_3_ [32]. By looking at the pH in the present experiment, it can be concluded that the majority of the NH_4_^+^-N was taken up by the duckweeds.

The chloride concentrations in the experiment increased with increasing ammonium supplement because ammonium chloride was partly used to increase the NH_4_^+^-N concentrations. Liu et al. [33] recommended an NaCl concentration below 75 mM for *L. minor* for N and P removal from water. Concentrations of 50 mM and higher caused a decrease in the fresh weight and chlorophyll content of *L. minor*. The maximum Cl^−^ concentration used in the presented experiment was 9.2 mM in [0-100]/50. Therefore, the significantly reduced RGRs for both species in the ratios [50-50], [25-75], and [0-100], as compared to [75-25], could not be caused by the presence of chloride. 

Duckweeds (species not identified) grown in irrigation ponds in Jordan yielded an average CPC of 26% [34]. Mohedano et al. [35] investigated the CPC of duckweed species grown in anaerobically digested swine manure in two consecutive ponds. The average CPC in the primary pond was 35% (based on dry matter) and decreased to 28% in the secondary pond, which had less nutrients available. The estimated productivity of both ponds was 24 t year^−1^ ha^−1^ (ca. 46 g week^−1^ m^−2^). This value is slightly higher than our maximum value (45 g week^−1^ m^−2^). The lower CPC in their study was compensated for by a higher RGR (0.24 d^−1^). Chakrabarti et al. [4] reported a yield of 703 kg month^−1^ ha^−1^ *L. minor* (ca. 17.5 g week^−1^ m^−2^) with RGRs ranging between 0.073 d^−1^ and 0.422 d^−1^. The duckweed was cultivated on different media containing organic manure or inorganic fertilizers. The final CPC was 36.07% and 27.12% for duckweeds grown in organic manure and in inorganic fertilizer, respectively.

The modified Schenk-Hildebrandt medium used by Appenroth et al. [2] had a nitrate-N to ammonium-N ratio of roughly 90-10. The total ammonium-N concentration (1.3 mM) was about the same as in [75-25]/50 (1.24 mM) of the modified N-medium, while nitrate concentrations were higher in the modified Schenk-Hildebrand medium. The CPC in the presented investigation was above 25% for *L. minor* and above 35% for *W. hyalina* in almost all ratios and dilutions, which was also the result in Appenroth et al. [2] for both species. Only [100-0]/1 showed a lower value of 21.1% and 30.3% for *L. minor* and *W. hyalina*, respectively. The nitrogen availabilities in these two experiments were only slightly different, thereby confirming our own results. 

If duckweed should be cultivated in an agrarian system in order to produce food and feed in the future, a standardized cultivation process needs to be implemented to yield a standardized product quality. Of high interest concerning a standardized non-axenic cultivation process is the growth of algae and microorganisms and how they influence duckweed growth and culture medium composition. The use of plant growth-promoting bacteria in particular may open up new opportunities [36]. Alongside the quality, the amount of biomass and protein yielded is of great importance. The variation of the initial biomass, hence surface coverage, could have an important impact on the productivity of a system. The higher the initial biomass, the higher the nutrient requirement over time. Therefore, highly diluted nutrient media result in low growth rates. An initial surface coverage of 20% seems optimal for a high RGR [37,38]. Such a low initial duckweed biomass, however, means less competition for other organisms competing for nutrients and light. Therefore, in these experiments, an initial surface coverage of 60% was selected. To avoid growth inhibition due to high densities (“overcrowding” [39]), a regular harvest interval should be defined. Regarding the protein yield, the RPY should be considered a good indicator for the productivity of a duckweed system.

## 5. Conclusions

Implementing conditions that increase the RGR and CPC, positively affect the RPY. One such condition is a suitable nutrient composition of standardized quality. The concentration of nutrients in the medium, as well as the ratio between nitrate-N and ammonium-N, influenced the RGR, CPC, and RPY in the duckweeds *L. minor* and *W. hyalina*. The modification of the promising N-medium, with a substitution of 25% nitrate-N by ammonium-N at 50% dilution, significantly increased the RPY for both species when compared to the nitrate-only ratio at the same dilution. *L. minor* yielded 41.6 ± 2.2 g week^−1^ m^−2^, while *W. hyalina* reached 45.0 ± 5.7 g week^−1^ m^−2^. 

However, other abiotic factors, such as light intensity, light spectrum, photoperiod, temperature, water and duckweed movement, as well as biotic factors, such as the growth of algae and microorganisms and their effects on duckweed, should be closely investigated. A stable cultivation process is only possible if all the biotic and abiotic factors are complementary and optimized for the species of choice.

## Figures and Tables

**Table 1:** Formulation of seven stock solutions (g L^−1^) for five different nitrate-N to ammonium-N ratios ([100-0], [75-25], [50-50], [25-75], and [0-100]), based on the N-medium.

Stock Solution	Product Name	Main Components	[100-0] (g L^−1^)	[75-25] (g L^−1^)	[50-50] (g L^−1^)	[25-75] (g L^−1^)	0-100 (g L^−1^)
1	Calcinit	NO_3_^−^-N, NH_4_^+^-N, Ca^+^	47.2	35.4	23.6	11.8	0
1	Krista K Plus	NO_3_^−^-N, K^+^	161.8	121.3	80.9	40.4	0
2	NH_4_Cl	NH_4_^+^-N, Cl^−^	0	0	26.7	53.5	80.2
3	OCI Granular 2	NH_4_^+^-N, SO_4_^2−^	0	33	33	33	33
4	KCl	K^+^, Cl^−^	0	29.8	59.6	89.5	119.3
4	CaCl_2_ · 2 H_2_0	Ca^+^, Cl^−^	0	7.4	14.7	22.1	29.4
5	Krista MKP	PO_4_^3−^, K^+^	27.2	27.2	27.2	27.2	27.2
6	Epso Combitop	Mg^2+^, SO_4_^2−^, Mn^2+^, Zn^2+^	49.3	49.3	49.3	49.3	49.3
6	Borax	BO_3_^3−^	0.06	0.06	0.06	0.06	0.06
6	Mangaan	Mn^2+^, SO_4_^2−^	0.44	0.44	0.44	0.44	0.44
6	MoNa_2_O_4_ · 2 H_2_0	MoO_4_^2−^, Na^+^	0.02	0.02	0.02	0.02	0.02
7	Ferty 72	Fe^3+^	2.2	2.2	2.2	2.2	2.2

**Table 2:** Nutrient composition (mM) of the modified N-media with five different NO_3_^−^-N (light grey) to NH_4_^+^-N (dark grey) ratios at an initial concentration of 100%. These concentrations were diluted to the final concentrations of 10% and 50%, and, in some cases, to 1% and 5%.

NO_3_^−^-N to NH_4_^+^-N Ratio Substance	[100-0] (mM)	[75-25] (mM)	[50-50] (mM)	[25-75] (mM)	0-100 (mM)
NO_3_^−^-N	10.1	7.6	5.1	2.6	0.1
NH_4_^+^-N	0	2.5	5	7.5	10
PO_4_^3−^	1	1	1	1	1
K^+^	9.1	9.1	9.1	9.1	9.1
Mg^2+^	1.3	1.3	1.3	1.3	1.3
SO_4_^2−^	2.0	3.2	3.2	3.2	3.2
Ca^+^	2.2	2.2	2.2	2.2	2.2
Cl^−^	0.9	3.4	8.4	13.4	18.4
Fe^3+^	0.025	0.025	0.025	0.025	0.025
BO_3_^3−^	0.005	0.005	0.005	0.005	0.005
Mn^2+^	0.013	0.013	0.013	0.013	0.013
Zn^2+^	0.01	0.01	0.01	0.01	0.01
MoO_4_^2−^	0.0004	0.0004	0.0004	0.0004	0.0004
Na^+^	0.8	0.8	0.8	0.8	0.8

**Table 3:** Measured concentrations of NO_3_^−^-N and NH_4_^+^-N (mg L^−1^) and EC-values (mS cm^−1^) at the start of the experiment in the different nutrient media. For the composition of the undiluted nutrient medium, see Table 2. The diluted nutrient media contain 1, 5, 10, or 50% of the undiluted medium.

Ratio	[100-0]				[75-25]		[50-50]		[25-75]		0-100	
Dilution (%)	1	5	10	50	10	50	10	50	10	50	10	50
NO_3_^−^-N (mg L^−1^)	2.7	8.8	15.3	71.2	12.1	56.7	10.1	35.3	5.2	16.6	1.1	1.4
NH_4_^+^-N (mg L^−1^)	0.06	0.17	0.29	1.1	3.6	17.3	7.1	32.5	10.7	51.3	14.4	64.4
EC (mS cm^−1^)	0.43	0.46	0.53	1.15	0.6	1.33	0.66	1.58	0.65	1.64	0.64	1.84

## Supplementary Materials

The following are available online at https://www.mdpi.com/article/10.3390/plants10081741/s1, Figure S1: relative weekly yield (RY, week^−1^) based on DW, for *L. minor* (grey shaded columns) and *W. hyalina* (white columns), cultivated for seven days in nutrient solutions with different NO_3_^−^-N to NH_4_^+^-N ratios in different dilutions, based on N-medium. For further explanations, see Figure 1. Table S1: tap water analysis municipal utilities Osnabrueck Wittefeld.
