# Supplementary material for: Influence of the Nitrate-N to Ammonium-N Ratio on Relative Growth Rate and Crude Protein Content in the Duckweeds Lemna minor and Wolffiella hyalina
Source: Plants (Basel). 2021 Aug 23;10(8):1741. doi: 10.3390/plants10081741 (PMC8399352; doi:10.3390/plants10081741)
Supplement: Supplementary file 1 [file plants-10-01741-s001.zip › Figure S1 - relative weekly yield.pdf]

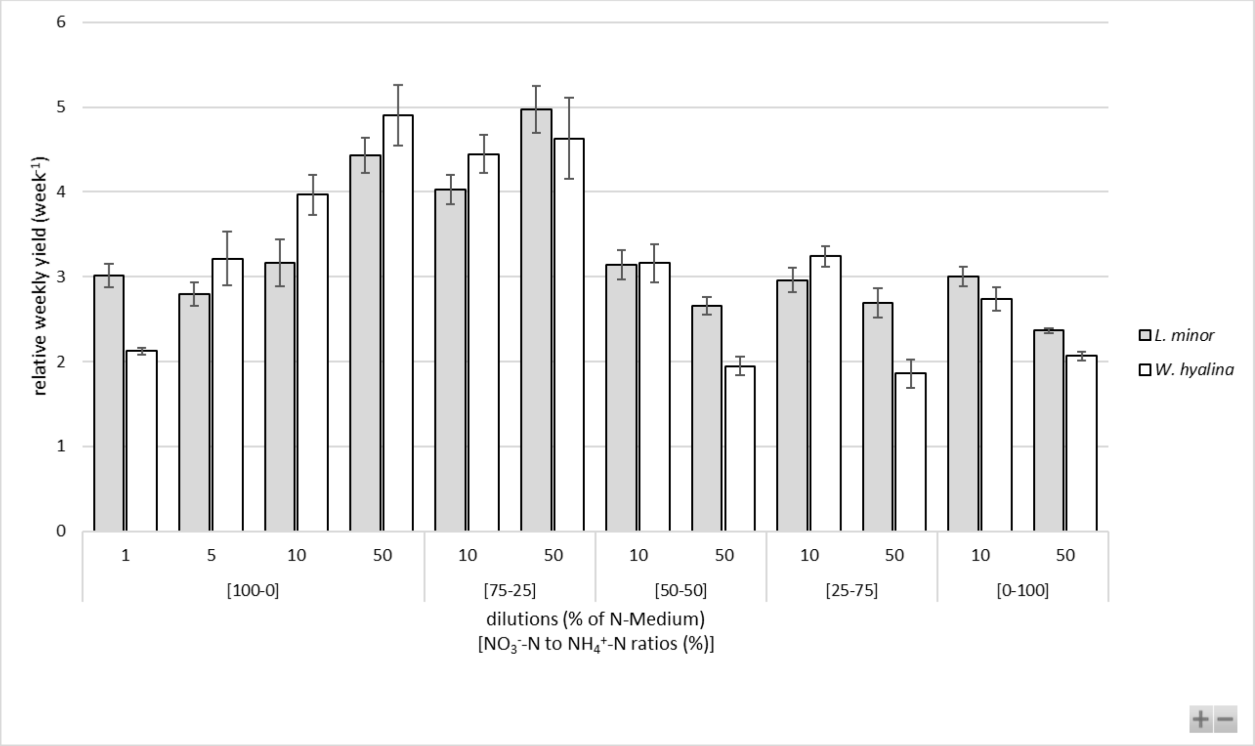

Figure S1: relative weekly yield (RY, $week^{-1}$) based on DW, for *L. minor* (grey shaded columns) and *W. hyalina* (white columns), cultivated for seven days in nutrient solutions with different NO_3^--N to NH_4^+-N ratios in different dilutions, based on N-medium. For further explanations, see Fig. 1.
