# Supplementary material for: Influence of the Nitrate-N to Ammonium-N Ratio on Relative Growth Rate and Crude Protein Content in the Duckweeds Lemna minor and Wolffiella hyalina
Source: Plants (Basel). 2021 Aug 23;10(8):1741. doi: 10.3390/plants10081741 (PMC8399352; doi:10.3390/plants10081741)
Supplement: Supplementary file 1 [file plants-10-01741-s001.zip › Table S1- tap water analysis municipal utilities Osnabrueck Wittefeld.pdf]

Table S1: tap water analysis municipal utilities Osnabrueck Wittefeld

substance	unit	german drinking water ordinance limits	measured concentration
boron	mg L^{-1}	1	0.02
nitrate	mg L^{-1}	50	7.7
ammonium	mg L^{-1}	0.5	<0.02
chloride	mg L^{-1}	250	32
iron	mg L^{-1}	0.2	0.014
manganese	mg L^{-1}	0.05	-
sodium	mg L^{-1}	200	17.4
sulfate	mg L^{-1}	250	94
potassium	mg L^{-1}	-	3.26
calcium	mg L^{-1}	-	49.5
magnesium	mg L^{-1}	-	7.5
